# In situ Gelling Hydroxypropyl Cellulose Formulation Comprising Cannabidiol-Loaded Block Copolymer Micelles for Sustained Drug Delivery

**DOI:** 10.3390/ijms242216534

**Published:** 2023-11-20

**Authors:** Katya Kamenova, Denitsa Momekova, Georgy Grancharov, Anna Prancheva, Natalia Toncheva-Moncheva, Ervin Ivanov, Spiro Konstantinov, Petar D. Petrov

**Affiliations:** 1 Institute of Polymers, Bulgarian Academy of Sciences, 1113 Sofia, Bulgaria; kkamenova@polymer.bas.bg (K.K.); granchar@polymer.bas.bg (G.G.); a_prancheva@polymer.bas.bg (A.P.); ntoncheva@polymer.bas.bg (N.T.-M.); 2 Faculty of Pharmacy, Medical University of Sofia, 1000 Sofia, Bulgaria; dmomekova@pharmfac.mu-sofia.bg (D.M.); ervin.ivanov@gmail.com (E.I.); skonstantinov@pharmfac.mu-sofia.bg (S.K.); 3 Pobelch Gle Ltd., 1618 Sofia, Bulgaria

**Keywords:** in situ gels, cannabidiol, hydroxypropyl cellulose, polymeric micelles

## Abstract

Cannabidiol (CBD) is a natural terpenophenolic compound with known pharmacological activities, but the poor solubility of CBD in water limits its widespread use in medicine and pharmacy. Polymeric (nano)carriers demonstrated high potential for enhancing the solubility and therapeutic activity of lipophilic drugs such as CBD. Here, we report the elaboration of a novel hydroxypropyl cellulose (HPC)-based in situ gelling formulation for controlled delivery of CBD. In the first stage, nanosized polymeric micelles from poly(ethylene oxide)-*block*-poly(α-cinnamyl-ε-caprolactone-*co*-ε-caprolactone) (PEO-*b*-P(CyCL-*co*-CL) diblock copolymers) were used to increase the solubility of CBD in water. Different copolymers were assessed, and the carrier with the highest encapsulation efficiency (EE) and drug loading capacity (DLC) was selected for further elaboration of nanocomposite in situ gel formulations. Next, the sol-to-gel transition behavior of HPC as a function of K_2_SO_4_ concentration in the aqueous solution was investigated by microcalorimetry and dynamic oscillatory rheology, and the optimal formulation capable of forming a physical gel under physiological conditions was determined. Finally, injectable nanocomposite hydrogels comprising cannabidiol were fabricated, and their drug release profile and cytotoxicity against human tumor cell lines were evaluated. The in situ gels exhibited prolonged drug release over 12 h, controlled by gel erosion, and the cytotoxicity of formulated cannabidiol was comparable with that of a free drug.

## 1. Introduction

Hydrogels are a unique class of polymeric materials capable of retaining large amounts of water while maintaining their integrity and shape [1,2]. In fact, these are three-dimensional, physically or chemically crosslinked polymeric networks of natural or synthetic origin, swollen in water [3]. The specific features of hydrogels, such as biocompatibility, softness, flexibility, porosity, high permeability, etc., make them attractive for biomedical applications as they resemble the characteristics of the native extracellular matrix. Hence, research on hydrogels intended for tissue engineering and the delivery of therapeutic agents has steadily increased over the last few decades. Overall, the chemically crosslinked hydrogels are tough and elastic and thus satisfy the highly demanded properties in dynamic environments such as cartilage, skin, and cardio-related devices. In turn, physically crosslinked hydrogels can be easily prepared without any chemical reaction, which facilitates the manipulations and ensures safety in in vivo conditions [4]. In this case, the polymer network is formed because of hydrophobic, ionic, or host-guest interactions, hydrogen bonding, self-assembly, complexation, etc. The physical gelation is a reversible process, and the corresponding hydrogel can be dissolved by changing the environmental conditions [5].

Recently, research on hydrogels for biomedical applications has focused on stimuli-responsive systems that can form gel at physiological conditions and thus reduce the risks involved in the surgical implantation of conventional hydrogels [6]. In this aspect, temperature-responsive hydrogels are among the most exploited platforms for developing in situ gel-forming formulations [4,7]. Such systems undergo a physical phase (sol-gel) transition upon temperature changes, allowing easy administration by applying the liquid form with a conventional syringe even within the surgery area for tumor resection and subsequent gelation in situ at physiological temperature. Indeed, injectable temperature-responsive gels have been extensively investigated as carriers of therapeutic agents such as drugs, growth factors, enzymes, wound dressing, etc. [8,9,10,11]. 

Polymers from natural sources such as polysaccharides (hyaluronic acid, alginate, cellulose, chitosan, dextran, starch, etc.) and proteins (gelatin, fibrin, and collagen) have been widely used for fabricating injectable hydrogels [6,12]. Natural materials are more advantageous than synthetic ones because of their biocompatibility, lack of toxicity, better interaction with the surrounding tissues, and biodegradability. However, only several natural polymers (methylcellulose, hydroxypropyl cellulose, hydroxypropyl methylcellulose, xyloglucan, and gelatine) can undergo thermo-reversible gelation in aqueous media, and none of them exhibits a sol-gel transition temperature (T_sol-gel_) close to physiological temperature [2,13]. For example, gelatine, which belongs to the polymers having an upper critical solution temperature (UCST), can form a physical hydrogel below 25 °C, but at 37 °C, this gel dissolves [13]. Contrariwise, hydroxypropyl methylcellulose, a polymer with a lower critical solution temperature (LCST), is soluble in water at lower temperatures and tends to form hydrogels above 65 °C [14]. Generally, two different strategies have been applied to tune the T_sol-gel_ of cellulose derivatives and thus achieve gelling material at the desired temperature of 37 °C. The first approach is based on chemical modification, where synthetic (co)polymers or units are covalently attached to the polysaccharide macrochains. For instance, Miao et al. prepared a thermoresponsive hydrogel via click reactions between alkynyl-functionalized hydroxypropyl cellulose and azide-modified poly(N-isopropylacrylamide-co-hydroxyethyl methylacrylate polycaprolactone) [15]. This system forms hydrogel at approximately 30 °C, while the T_sol-gel_ of pure HPC is 44 °C. Another study revealed that the reaction of HPC with N-methyl carbamoylimidazole can also decrease the LCST of HPC, and T_sol-gel_ is dependent on the methylcarbamate degree of substitution [16]. It should be noted that multistep chemical reactions and accompanying purification procedures may be considered a disadvantage for practical use. Adding salts, which can decrease the solubility of polymers in water (the salting-out effect), seems like a more facile strategy for reducing the T_sol-gel_ of temperature-responsive cellulose derivatives [14,17]. By this way, one can easily formulate a thermoresponsive polysaccharide drug delivery system capable of forming in situ gels under physiological conditions. 

Cannabidiol is a natural terpenophenolic compound with proven pharmacological activities such as anti-inflammatory, antioxidant, analgesic, antidepressant, etc. [18]. One of the most perspective-oriented directions of cannabidiol research is its assessment as an anti-cancer agent [19]. However, the poor solubility of CBD in water limits its widespread use for this purpose due to low bioavailability and unfavorable biodistribution [20]. In particular, the studies dealing with hydrogel formulations of CBD for cancer treatment are very limited. Very recently, we reported a nanocomposite hydrogel based on CBD-loaded polymeric micelles embedded in a chemically crosslinked hydroxyethyl cellulose cryogel matrix [21]. Using polymeric micelles, the solubility of CBD in water was improved while achieving a sustained release of CBD from the cryogel carrier and pronounced cytotoxicity against human tumor cells. In the present work, a novel in situ gelling formulation based on HPC and CBD-loaded PEO-*b*-P(CyCL-*co*-CL) micelles was developed, and its antiproliferative potential against human breast cancer MCF-7cells was tested in a comparative way vs. a free drug in order to enable sustained CBD release after application in the surgery area of the primary tumor and the dissected lymph nodes. In addition, the biocompatibility of the developed nanocomposite gel carriers was investigated on normal fibroblast cells and was shown to be devoid of significant cytotoxic potential. To get a physical hydrogel at physiological temperature, the T_sol-gel_ of HPC was decreased by adding K_2_SO_4_ to the polymer solution. At a salt concentration of 0.15 molL^−1^, the sol-gel transition point of the system was adjusted to approximately 35 °C, as confirmed by microcalorimetry and dynamic rheological measurements. 

## 2. Results and Discussion

### 2.1. Preparation of Cannabidiol-Loaded Polymeric Micelles

**Figure 1:**
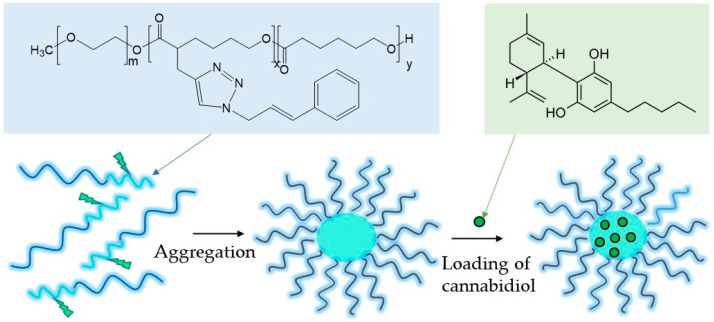
Sketch of the formation of cannabidiol-loaded micelles via aggregation of PEO-*b*-P(CyCL-*co*-CL) diblock copolymer in water.

Nanosized micelles comprising a hydrophobic PCL-based core and a hydrated PEO shell were formed via the aggregation of amphiphilic PEO-b-P(CyCL-co-CL) and PEO-b-PCL diblock copolymers in water and then loaded with CBD (Figure 1, Table S1). 

PEO-b-PCL block copolymers have been commonly used for biomedical applications and fabricating nanocarriers intended for controlled release of hydrophobic drugs [22]. The incorporation of cinnamyl groups in the core-forming PCL block was previously found to enhance the compatibility between the hydrophobic drug caffeic acid phenethyl ester and the micellar core, resulting in increased encapsulation efficiency and drug loading capacity of the systems [23]. In this study, two types of carriers from cinnamyl-modified and unmodified copolymers were tested for solubilization and release of CBD. Well-defined micellar nanocarriers were prepared by the solvent evaporation method [24]. Since the used copolymers were not directly soluble in water, they were first dissolved in acetone, which is a common solvent for PEO and PCL, and the copolymer solution was slowly added to distilled water. The organic solvent was evaporated under vacuum to afford transparent micellar solutions of concentration 1 gL^−^^1^. The copolymer concentration was approximately an order of magnitude higher than the critical micelle concentrations (CMC) of PEO-b-P(CyCL-co-CL) and PEO-b-PCL, as determined in our previous study [24]. Next, the micelles were loaded with CBD at an initial copolymer/drug mass ratio of 10:1, 7:1, and 5:1. CBD was dissolved in ethanol before being added to the aqueous micellar solution. Finally, the organic solvent was evaporated under vacuum, and the drug-loaded micelles were characterized and used in our further experiments for fabricating nanocomposite in situ gels.

**Figure 2:**
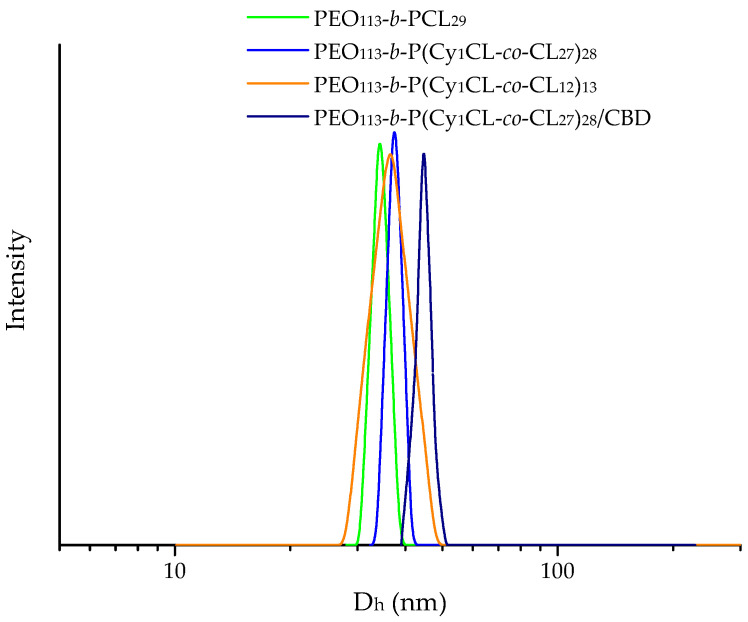
Hydrodynamic diameter distribution of blank and drug-loaded micelles (copolymer/CBD mass ratio 10:1) in water.

Dynamic and electrophoretic light scattering methods were employed to determine the size distribution, hydrodynamic diameter (D_h_), and zeta potential of the blank and CBD-loaded micelles (Table 1). All micelles exhibited a monomodal particle size distribution, as illustrated in Figure 2. The mean D_h_ varied between 34 and 44 nm, and the particle surface charge was slightly negative, depending on the formulation. It should be noted that the characteristics of modified and unmodified copolymer nanocarriers were identical. The loading of CBD caused a negligible increase in the micellar size, which is probably due to the superior structural stability of PCL-based nanocarriers. Under the reported experimental conditions, the micelles are kinetically frozen structures that cannot undergo notable rearrangements during the loading procedure [23,24]. The drug-loaded micelles exhibited higher negative values of zeta potential as compared to the blank ones. In addition, the PEO-b-PCL micelles do not dissociate rapidly upon changes in environmental conditions (temperature, concentration, etc.), which is important for preventing the burst release of the drug [24]. Therefore, PEO-b-PCL nanocarriers were considered advantageous for embedding into a hydrogel matrix. The morphology of blank and CBD-loaded micelles was visualized by Atomic Force Microscopy (AFM) analyses. The two systems exhibited identical nanosized particles of spherical shape (Supplementary Figure S1). These results were consistent with the DLS data reported above.

The carriers based on PEO-*b*-P(CyCL-*co*-CL) and PEO-*b*-PCL diblock copolymers demonstrated relatively high DLC and EE, which were dependent on the copolymer composition and copolymer/drug mass ratio (Table 2). Definitely, the EE and DLC of cinnamyl-modified copolymer carriers were higher compared to the corresponding unmodified analogue (PEO_113_-*b*-P(CyCL_1_-*co*-CL_27_)_28_ vs. PEO_113_-*b*-PCL_29_). These results confirmed our hypothesis that the cinnamyl groups enhance the compatibility between the micellar core and CBD and thus improve the loading capacity of the carrier. On the other hand, a dependence was found between the EE and DLC of the two cinnamyl-modified copolymers and the length of the PCL chain (degree of polymerization 27 vs. 12), indicating that the more hydrophobic copolymer could encapsulate a larger amount of CBD. The highest DLC values for the three investigated systems were obtained at the lowest copolymer/drug mass ratio (5:1), while the highest EE values were calculated at the highest copolymer/drug mass ratio (10:1).

### 2.2. Gelation of Hydroxypropyl Cellulose in Water in the Presence of K_2_SO_4_

**Figure 3:**
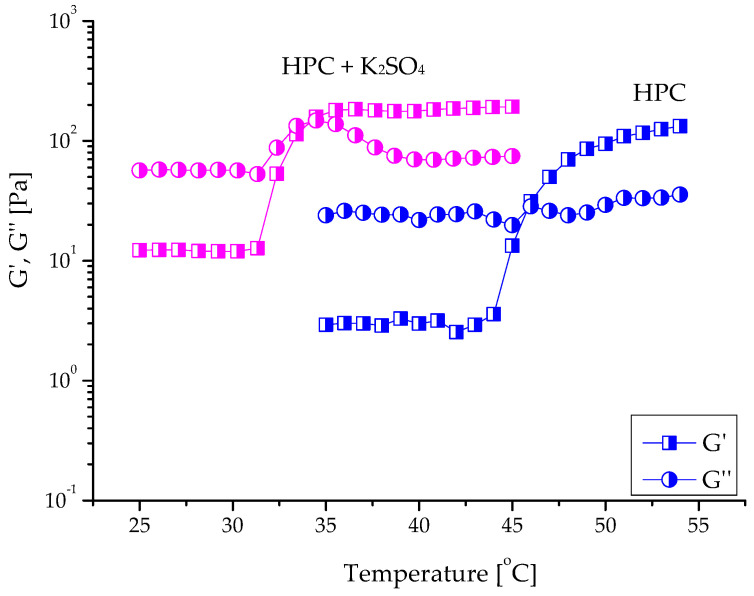
Temperature dependence of the storage and loss moduli of HPC dissolved in water (10 mass%) without salt (blue) and in the presence of 0.15 molL^−1^ K_2_SO_4_.

As mentioned before, HPC is a temperature-responsive natural polymer possessing a lower critical solution temperature of about 44–45 °C, which is above the body temperature (37 °C) [16]. However, for biomedical applications of in situ gel forms, the phase (sol-to-gel) transition must be shifted to lower temperatures, usually in the range between 33 and 35 °C [25]. Our preliminary tests with microcalorimetry showed that the dissolved HPC in distilled water exhibits a phase transition at a temperature of 46.4 °C (Supplementary Figure S2). Further studies of concentrated HPC aqueous solutions (10, 12, and 15 mass%) by oscillatory shear rheometry revealed a crossover of the elastic and loss moduli (G′ = G″) at approximately 46 °C. The profiles of the sol-gel transition curves of samples containing 10 (illustrated in Figure 3), 12, and 15 mass% HPC were identical. 

Adding salts to aqueous solutions of thermosensitive polymers is among the most favored strategies used to shift the phase transition of the polymer system to lower temperatures [17]. In this study, the ability of K_2_SO_4_ to decrease the sol-gel transition temperature (T_sol-gel_) of HPC in distilled water was investigated in detail. The measurements were conducted at an oscillatory frequency (f) of 1 Hz and a strain amplitude (γ_0_) of 0.001, which is inside the linear viscoelastic regime (Supplementary Figure S3). The polymer (10, 12, and 15 mass%) was dissolved in water containing different concentrations of salt (0.1–0.3 molL^−1^) at 25 °C, and the physical gelation of different systems as a function of temperature was monitored. At low temperatures, the samples exhibited typical polymer solution behavior. The loss modulus (G″) was higher than the storage modulus (G′), indicating that the viscous component was predominant over the elastic one. Above the given temperature, the two moduli tended to increase, with a more pronounced effect for G′. At a certain point, G′ and G” crossed (the sol-gel transition point), and then the curves acquired the typical pattern for gels where G′ > G″ (Figure 3).

**Figure 4:**
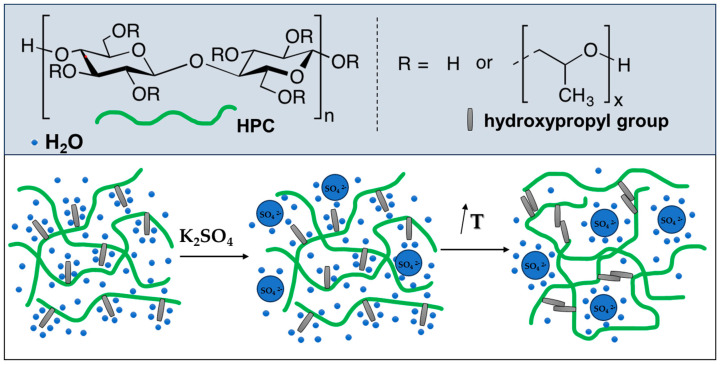
Schematic representation of K_2_SO_4_-assisted gelation of HPC in water.

The salting-out phenomenon in water-based polymer systems is related to changes in the structuring of water when a salt is added. The effects of ions on water structure are attributed to the competition between ion-water and water-water interactions. Multivalent anions such as SO_4_^2−^ have a strong ability to compete for water molecules in a polymer solution [17]. They cause a strong electrostatic orientation of water molecules around the anions, making the water structure more ordered. As a result of adding K_2_SO_4_, fewer water molecules were free to solvate the polymer chains, which facilitated the hydrophobic interaction of the hydroxypropyl groups and led to the formation of HPC gel at a lower temperature (Figure 4).

**Figure 5:**
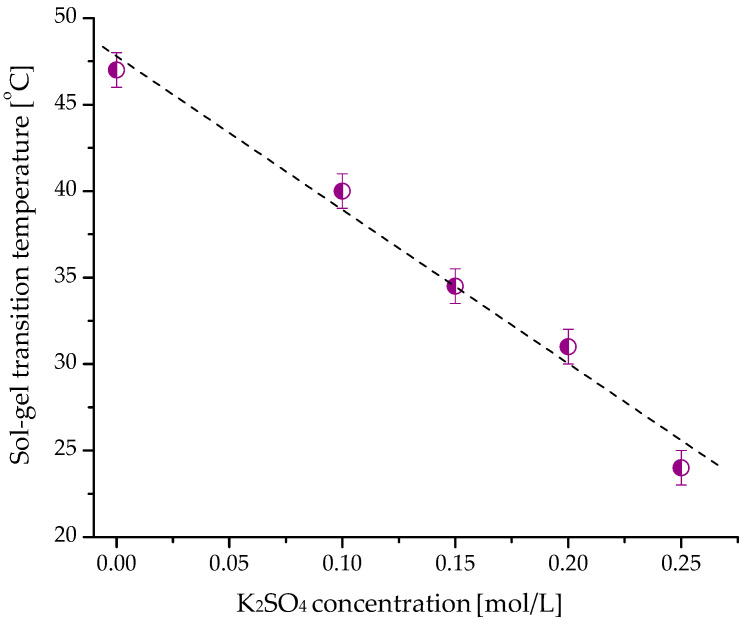
Effect of K_2_SO_4_ concentration on the sol-gel transition of HPC (10 mass%) in water.

This assumption was supported by experimental results revealing a linear decrease in T_sol-gel_ with increasing concentrations of K_2_SO_4_ in the samples (Figure 5). The higher the amount of SO_4_^2−^ anions, the lower the T_sol-gel_. The rheological data were in good agreement with the results from nanoDSC analyses (Supplementary Figure S2). Indeed, a temperature shift from 46 to 35 °C was determined by both methods when K_2_SO_4_ (0.15 molL^−1^) was added to the HPC solution. Adding K_2_SO_4_ also affected, to some extent, the elastic properties of hydrogel. As can be seen from the data in Table 3, at equal polymer concentrations, the hydrogel formed in the presence of salt exhibited a slightly higher storage modulus than the hydrogel obtained in pure water. 

The polymer concentration, ranging from 10 to 15 mass%, did not significantly influence the sol-gel transition point, indicating that the gelation under the reported conditions was predominantly a temperature-driven process. On the other hand, the increase in HPC concentration yielded hydrogel with notably enhanced elastic properties (Table 3). Regarding the potential application of HPC in situ gels as drug carriers, an optimal K_2_SO_4_ concentration of 0.15 molL^−1^ was chosen for our further experiments.

### 2.3. Fabrication of Injectable Nanocomposite In Situ Hydrogels Comprising Cannabidiol

**Figure 6:**
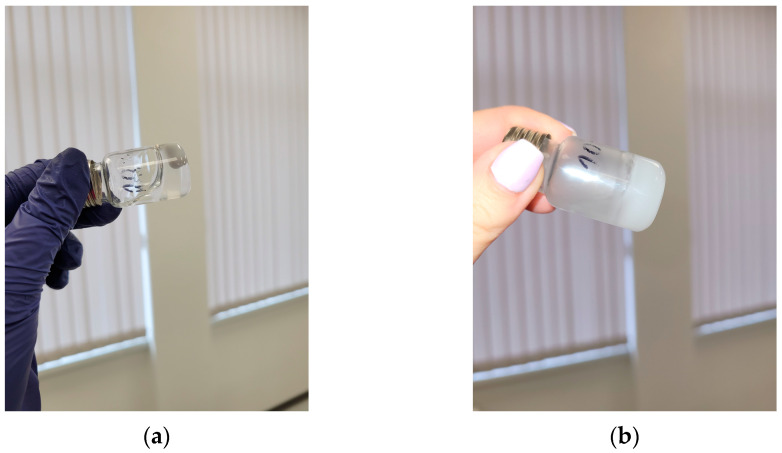
A viscous solution of HPC, K_2_SO_4_, and CBD-loaded micelles in water at 25 °C (**a**) and the corresponding physical gel at 37 °C (**b**).

Nanocomposite HPC in situ hydrogels, containing CBD-loaded micelles, were fabricated by dissolving HPC, K_2_SO_4_, and nanoformulated PEO_113_-b-P(CyCL_1_-*co*-CL_27_)_28_/CBD in water at 25 °C and subsequent heating to 37 °C (Figure 6). Three different concentrations of HPC (10, 12, and 15 mass%) were used, while the concentrations of salt (0.15 molL^−1^) and micelles (1 gL^−1^) were kept constant. As discussed earlier, the physical gel was formed as a result of hydrophobic interactions between pendant hydroxypropyl groups in the polysaccharide chains. The presence of SO_4_^2−^ anions in the system facilitated this process and decreased the sol-gel transition temperature below the body temperature. Adding drug-loaded micelles had only a minor effect on the rheological properties of HPC/K_2_SO_4_ systems.

**Figure 7:**
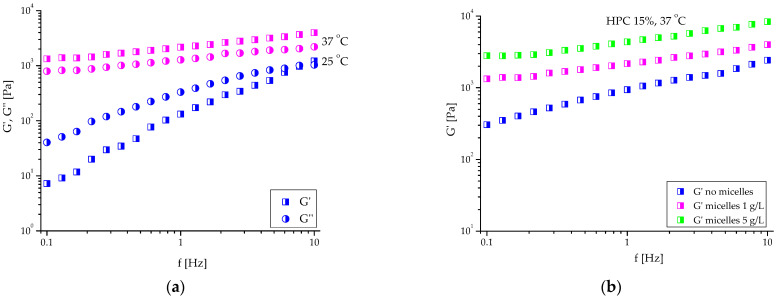
Variation in: (**a**) storage and loss moduli as a function of the oscillatory frequency for HPC system (15 mass%), containing K_2_SO_4_ (0.15 molL^−1^) and CBD-loaded micelles (1 gL^−1^), measured at 25 and 37 °C, and (**b**) storage modulus as a function of the oscillatory frequency at 37 °C for HPC hydrogels, formed with K_2_SO_4_ (0.15 molL^−1^), without micelles and with two different concentrations of CBD-loaded micelles (1 and 5 gL^−1^).

Dynamic rheological studies confirmed that the elaborated dosage forms are liquid at room temperature and physical gels at 37 °C, respectively (Figure 7a). The variation of G′ and G″ at 25 °C is frequency-dependent, and G″ > G′ in the investigated range. In contrast, at 37 °C, the storage modulus for the formulations was significantly higher than the loss modulus, and the dependence of two moduli on the oscillatory frequency was less pronounced. Moreover, G′ values measured at 37 °C were one order of magnitude larger than G′ at 25 °C, indicating the formation of a highly elastic material. Embedding micelles in HPC hydrogel did not change T_sol-gel_, but it contributed to the increase in the elastic modulus of the material. Indeed, G′ of the nanocomposite hydrogel was more than two times higher than G′ of the blank gel obtained at the same conditions (Figure 7b). Such reinforcing effect of micelles we have previously found for nanocomposite HPC cryogel comprising crosslinked PEO-*b*-PPO-*b*-PEO micelles with a rigid core [26]. In the case of PEO-*b*-P(CyCL-*co*-CL) micelles, the PCL-based core is rigid at 37 °C, and we hypothesized that this feature of the nanocarriers is the main factor in increasing the stiffness of the material. Having in mind that the mass ratio of HPC/copolymers in the studied sample was 150:1, we prepared an in situ gel with five times more concentrated sample of CBD-loaded micelles and then performed rheological measurements (Figure 7b). The results undoubtedly confirmed our assumption that the increase of G′ is due to the presence of PEO-*b*-P(CyCL-*co*-CL) micelles. In fact, the G′ values at 1 Hz for pure HPC hydrogel and nanocomposite HPC hydrogels, containing 1 and 5 gL^−1^ CBD-loaded micelles, were 940, 2165, and 4360 Pa, respectively.

It should be noted that the transition from viscous liquid to hydrogel was completed in approximately 10 s when the sample was heated on the rheometer plate from 25 to 37 °C (Supplementary Figure S4). During the process, some free water was released. Upon cooling, the system reversibly formed sol within the same time interval. 

In general, the rheological behavior of the developed HPC-based drug formulations fulfills one of the most important requirements for injectable hydrogels—the formulation is viscous at room temperature and forms an elastic gel in situ at body temperature. This characteristic, together with the significantly improved solubility of CBD in aqueous media (with the aid of micelles), makes the nanocomposite HPC in situ gels a promising platform for developing advanced injectable forms of CBD with specific locoregional antineoplastic activity.

### 2.4. Drug Release Study

**Figure 8:**
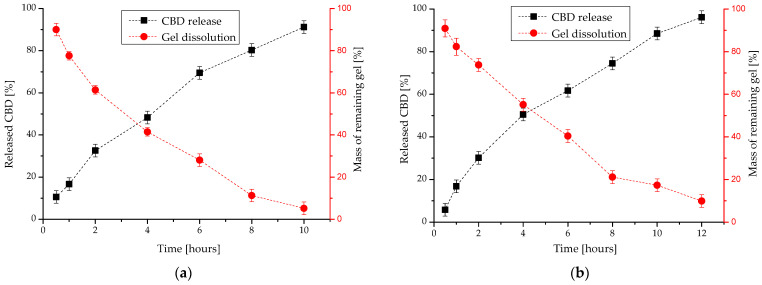
In vitro cannabidiol release and gel dissolution profiles of nanocomposite thermosensitive in situ gels based on HPC (15 mass%) and CBD-loaded PEO_113_-*b*-P(CyCL_1_-*co*-CL_27_)_28_ micelles containing: (**a**) 0.1 gL^−1^ CBD and (**b**) 0.5 gL^−1^ CBD (polymer/drug mass ratio 10:1).

The release profiles of cannabidiol from the developed nanocomposite in situ gels were evaluated by a membrane-less method where the release medium is in direct contact with the gel surface. Such conditions resemble the in vivo conditions in the wound area, where gels can be potentially applied after surgical eradication of breast cancer. In this study, two HPC hydrogels (15 mass%), containing 1 and 5 gL^−1^ of CBD-loaded PEO_113_-b-P(CyCL_1_-*co*-CL_27_)_28_ micelles (polymer/drug mass ratio 10:1), were used. As evident from the released profiles shown in Figure 8, the developed gels are able to release the encapsulated cannabidiol in a controlled manner over a period of 10–12 h. The gel containing a higher micellar fraction (5 gL^−1^) showed a slower cannabidiol release compared to the analogous composition with a lower micellar content (1 gL^−1^). A comparison of the drug release profiles to the gel dissolution profiles showed a good correlation between the two processes. This indicates that the cannabidiol release mechanism is controlled to the greatest extent by the gel erosion process. Both the erosion of the gel and the release of cannabidiol are slower in the sample with the higher micellar fraction. This fact can be explained by the reinforcing effect of the micelles on the gel structure. These observations correlate well with the results from dynamic rheological studies, where the higher storage modulus is associated with a higher micellar content in the gel matrix (see Figure 7b).

### 2.5. Evaluation of Cytotoxicity of Cannabidiol-Loaded In Situ Thermoresponsive Gels

**Figure 9:**
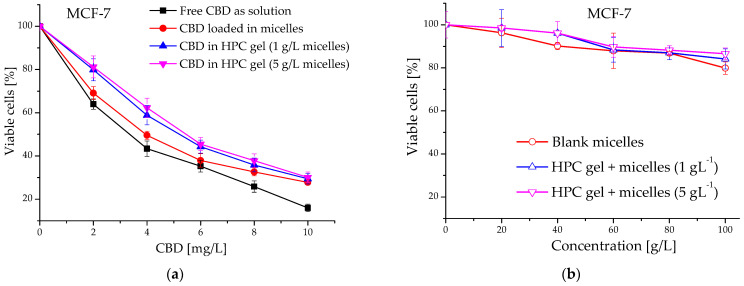
Concentration-response curves of (**a**) free CBD (as a solution in ethanol) and its formulations, and (**b**) blank micelles and the in situ nanocomposite HPC gels, against the human breast adenocarcinoma cell line (MCF-7).

**Figure 10:**
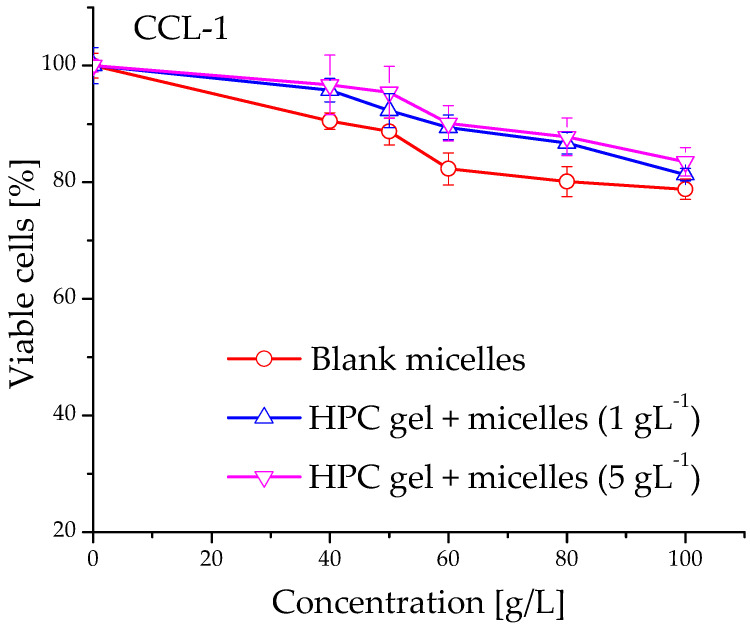
Viability of normal CCL-1 cells after exposure to blank PEO_113_-*b*-P(CyCL_1_-*co*-CL_27_)_28_ micelles and in situ nanocomposite HPC gels prepared thereof.

The anti-proliferative activity of blank and cannabidiol-loaded micelles and their in situ gel counterparts was evaluated in a comparative way vs. a free agent against malignant MCF-7 human tumor cells. The concentration-response curves are depicted on Figure 9a,b and the derived IC_50_ concentrations thereof are shown in Table 4. As is obvious from the presented data, the in vitro cytotoxicity of the CBD were influenced by the formulation properties. As expected, the free cannabidiol (applied from a stock solution of ethanol) showed the highest activity against MCF-7 cells in low micromolecular concentrations (IC_50_ = 10.7 µM). The two CBD formulations (micelles and nanocomposite in situ gels) showed slightly higher IC_50_ values as compared to the free drug (Table 4). In addition, shifts of the concentration-response curves to higher concentrations were found. Generally, the observed modulatory effect on CBD antitumor activity is possibly due to the slower CBD release from the in situ gel formulations (see Figure 8). As indicated, druG′s dissolution and diffusion in the gel structure are slowed down, resulting in lower concentrations of the free CBD in cell culture media and in the tumor cell vicinity, respectively. Our results correspond well to other published data about the cytotoxicity of nano-formulated CBD against similar breast cancer cell lines, namely breast cancer MCF-7 and MDA-MB-231 cells [27,28]. On the other hand, the results from the treatment of non-malignant fibroblast CCL-1 cells with non-loaded micelles and in situ gels do not show significant suppression of the vitality of the treated breast cancer and fibroblast cells. This result is a proof of the absence of cytotoxic potential in the developed carriers (Figure 9b and Figure 10).

Our experimental findings clearly indicate that the elaborated in situ gel formulation of cannabidiol preserves the antitumor efficacy of the drug and enables long-lasting delivery within the tumor environment. It can be assumed that CBD-loaded in situ gels may have beneficial advantages when applied in liquid form to the surgery area of the primary tumor and its metastatic spread to lymph nodes, liver, bones, etc.

## 3. Materials and Methods 

### 3.1. Materials

Hydroxypropyl cellulose (Klucel™ MF Pharm, Mw = 850,000 Da) was donated by Hercules Inc., Aqualon Division, Wilmington, NC, USA. Potassium sulphate (99%), ethanol (99.5%), methanol (≥99.8%), and acetone (99%) were purchased from Sigma-Aldrich via FOT, Sofia, Bulgaria, and used as received. Cannabidiol (purity > 95%, as determined by Gas Chromatography) was donated by PBG GLOBAL LTD., Sofia, Bulgaria. The block copolymers were synthesized as described elsewhere [24]. Synthetic details and characterization data are given in the ESI.

### 3.2. Preparation of Block Copolymer Micelles

The polymeric micelles were prepared by the solvent evaporation method. Briefly, 10 mg of each copolymer were dissolved in 5 mL of acetone, and the solutions were added dropwise to 10 mL of deionized water at 25 °C under stirring. The resulting solutions were stirred vigorously (800 min^−1^) at ambient temperature for 30 min. Subsequently, the organic solvent was evaporated under vacuum at 40 °C to afford stable micellar solutions with a concentration of 1 gL^−1^.

### 3.3. Loading of Cannabidiol into Micellar Carriers

A solution of CBD (1 gL^−1^) in ethanol was added dropwise to the aqueous micellar solutions (15 mL) at copolymer/CBD mass ratios of 10:1, 7:1, and 5:1. After stirring for 30 min, the ethanol was removed under vacuum at 40 °C. The micellar dispersions were filtered (0.45 µm), and the filter was rinsed with methanol. The amount of non-encapsulated CBD in the collected filter fraction was determined by UV-vis measurements (λ = 274 nm) using a calibration curve in methanol (Supplementary Figure S5). The encapsulation efficiency (EE) and drug loading capacity (DLC) were calculated from the equations:

$$EE\left(\%\right)=\frac{Total\;mass\;of\;CBD-Mass\;of\;free\;CBD}{Total\;mass\;of\;CBD}\;\times 100$$

$$DLC\left(\%\right)=\frac{Mass\;of\;CBD\;embedded\;into\;micelles}{Total\;mass\;of\;micelles}\;\times 100$$

### 3.4. Preparation of Blank HPC Hydrogels 

HPC hydrogels (10%, 12%, and 15% *w*/*v*) were prepared by dissolving HPC (0.4 g, 0.48 g, and 0.6 g) into 4 mL of pure water or aqueous solutions of K_2_SO_4_ (0.1, 0.15, 0.2, and 0.25 molL^−1^) under stirring at room temperature. After stirring for 20 min, the viscous solutions were kept overnight at room temperature (25 °C) before measurements. 

### 3.5. Preparation of Nanocomposite HPC Hydrogels

Nanocomposite formulations were prepared by dissolving HPC (0.6 g) in 4 mL of an aqueous solution of K_2_SO_4_ (0.15 molL^−1^) and CBD-loaded micelles (1 g/L^−1^) under stirring at room temperature. After stirring for 20 min, the viscous solutions were kept overnight at room temperature before measurements. 

### 3.6. Analysis

The hydrodynamic diameter of micelles was determined by using Zetasizer NanoBrook 90Plus PALS (Brookhaven Instruments, Holtsville, NY, USA), equipped with a 35 mW red diode laser (λ = 640 nm) at 25 °C and a scattering angle of 90°. The electrophoretic light scattering measurements were conducted on the same instrument at a scattering angle of 15° and 25 °C. The phase analysis light scattering (PALS) method was applied for measuring the electrophoretic mobility. Atomic force microscopy (AFM) height images were obtained with a Bruker Dimension Icon microscope (Bruker Corporation, Karlsruhe, Germany) in peak force tapping mode exploiting silicon nitride cantilevers with a spring constant of ~0.4 Nm^−1^. The ultraviolet-visible absorption spectra were recorded on a UV-vis spectrophotometer (Thermo Scientific, Waltham, MA, USA) using quartz cells with a path length of 1 cm. Calorimetric measurements were conducted using a nano-differential scanning calorimeter (nanoDSC 60200, TA Instruments, New Castle, DE, USA). Approximately 0.6 mL of sample solution and an equal amount of reference fluid (deionized water) were hermetically sealed into the sample and reference cells. DSC curves were recorded in the temperature range of 15 to 90 °C at a rate of 1 °Cmin^−1^. Dynamic rheological measurements were conducted with a HAAKE MARS 60 rheometer (Thermo Fisher Scientific, Waltham, MA, USA). The tests were performed with a parallel plate geometry (top plate diameter = 20 mm; gap = 1 mm) in controlled deformation (CD) mode. Three runs of each sample were conducted. Prior to the temperature sweep measurements, amplitude and frequency sweep tests were made to establish the linear viscoelastic range for HPC gels. The gelation process was studied in oscillation-temperature sweep experiments. Storage and loss moduli were determined at constant deformation and frequency (γ_0_ = 0.001; f = 1.000 Hz) in a given temperature range (25–45 or 35–55 °C). The sample were equilibrated for 60 s at the given temperature before recording a data point. 

### 3.7. In Vitro Drug Release

To evaluate the cannabidiol release profiles of optimally prepared nanocomposite in situ gels, a membrane-less diffusion method was utilized as described elsewhere [29] with slight modifications. Briefly, 1.5 mL of the thermosensitive solutions were placed into preweighed empty flat-bottom vials at room temperature, and then the vials were placed in a water bath at 37 °C until translucent solid gels were formed. Then, the weight of each vial plus the gel was measured on the balance, and the weight was recorded. Next, 2 mL of the release medium (PBS, pH 7.4, containing 5 mol% ethanol) pre-heated to 37 °C was carefully added over the surface of each gel, and the vials were transferred into a thermostatic shaker bath at 37 °C at 100 ± 10 rpm. At certain time intervals, the release medium from each vial was completely withdrawn from the containers and subjected to UV-VIS spectrophotometry for quantitative determination of the released cannabidiol (at λ = 274 nm), using a calibration curve in PBS + 5% ethanol (Supplementary Figure S6). The vials with the undissolved gel were dried and weighed to determine the weight fraction of the dissolved gel. The vials were then equilibrated at 37 degrees until a solid gel formed, and a 2 mL portion of fresh medium was added. The procedure was repeated several times until the gels were completely dissolved.

### 3.8. Evaluation of Cytotoxicity

#### 3.8.1. Cell Lines and Culture Conditions 

The in vitro cytotoxicity of elaborated empty micelles and nanocomposite in situ gels prepared thereof was evaluated on normal murine fibroblasts (CCL-1TM, NCTC clone 929, American Type Culture Collection—ATCC, Manassas, VA, USA), while the antiproliferative activity of free or formulated nanocomposite in situ gels cannabidiol was assessed in malignant human tumor MCF-7 cell line (breast cancer adenocarcinoma) obtained from ATCC, Manassas, VA, USA. The cells were cultivated according to the protocol instructions of the supplier and incubated under standard conditions at 37 °C in a 5% humidified CO_2_ atmosphere. CCL-1 cells were maintained following the recommendations of ISO 10993-5, Annex C (ISO 10993-5:2009, 2017) [30]. 

#### 3.8.2. MTT Colorimetric Assay 

The in vitro cytotoxicity of the elaborated nanocomposite in situ gels was evaluated using a standard MTT method with minor modifications [31]. The method measures the activity of mitochondrial enzymes in viable cells to reduce the yellow dye MTT (3- (4,5-dimethylthiazol-2-yl) -2,5-diphenyltetrazolium bromide) to violet formazan crystals. Exponentially growing cells were seeded in 96-well plates at a suitable density of 1.5 × 10^5^. After 24 h of incubation, cells were treated with serial dilutions of the tested formulations with respect to cannabidiol concentration. Following an exposure time of 72 h, sterilized MTT substrate solution (5 mg/mL in PBS) was added to each well, and the plates were further incubated for 1–4 h, allowing the formation of purple insoluble formazan crystals. The latter were dissolved in an isopropyl alcohol solution containing 5% formic acid, and the absorbance was measured at 550 nm. The collected absorbance values were blanked against MTT and isopropanol solutions and normalized to the mean value of the untreated control (100% cell viability). The experimental data were processed using a non-linear regression analysis algorithm, semi-logarithmic “dose-response” curves were constructed, and the corresponding half-inhibitory concentrations (IC_50_) were calculated.

## 4. Conclusions

A novel in situ gelling formulation for controlled delivery of CBD based on the natural polymer hydroxypropyl cellulose was developed. Nanosized polymeric micelles of PEO-*b*-P(CyCL-*co*-CL) diblock copolymers played a key role in achieving a water-soluble form of the lipophilic drug CBD. Embedding CBD into nanosized micellar carriers enabled the fabrication of a homogeneous viscous mixture of the temperature-responsive HPC, K_2_SO_4_, and nanoformulated CBD, which then formed a reversible physical gel. The salting-out effect of K_2_SO_4_ was exploited to tune the sol-gel transition of the HPC-based aqueous system at near body temperature. Thus, the transition from a liquid form to a gel at approximately 35 °C was completed with a K_2_SO_4_ concentration of 0.15 molL^−1^. Nanocomposite gels released the encapsulated cannabidiol in a controlled manner over a period of 10–12 h, which depends on the erosion time of the polymer matrix. Tests with non-malignant fibroblast CCL-1 cells revealed that the polymeric carriers themselves are not cytotoxic, while the CBD-loaded HPC hydrogels exhibited pronounced activity against malignant MCF-7 human tumor cells. The elaborated in situ gel formulation may have beneficial advantages when applied in liquid form to the surgery area of the primary tumor and its metastatic spread to lymph nodes, liver, bones, etc.

## Figures and Tables

**Table 1:** Hydrodynamic diameter and zeta potential of blank and CBD-loaded micelles in water.

Copolymer	D_h_ (nm)		ζ Potential (mV)	
	Blank	Loaded ^1^	Blank	Loaded ^1^
PEO_113_-*b*-PCL_29_	34 ± 3	37 ± 3	−4.7 ± 0.7	−11.3 ± 0.7
PEO_113_-*b*-P(CyCL_1_-*co*-CL_27_)_28_	37 ± 3	44 ± 4	−7.9 ± 0.9	−12.3 ± 0.6
PEO_113_-*b*-P(CyCL_1_-*co*-CL_12_)_13_	36 ± 2	40 ± 4	−3.9 ± 0.6	−5.2 ± 0.4

^1^ Copolymer/CBD mass ratio 10:1; the values of the other two ratios (5:1 and 7:1) were identical and within the given error.

**Table 2:** Drug loading capacity and encapsulation efficiency of the diblock copolymer micellar nanocarriers.

Formulation	Mass Ratio	DLC (%)	EE (%)
PEO_113_-*b*-PCL_29_/CBD	10:1	8 ± 0.7	82 ± 3
	7:1	9 ± 0.8	67 ± 3
	5:1	13 ± 0.7	66 ± 4
PEO_113_-*b*-P(CyCL_1_-*co*-CL_27_)_28_/CBD	10:1	9 ± 0.7	89 ± 3
	7:1	11 ± 0.7	83 ± 4
	5:1	16 ± 0.8	82 ± 3
PEO_113_-*b*-P(CyCL_1_-*co*-CL_12_)_13_/CBD	10:1	7 ± 0.7	75 ± 3
	7:1	10 ± 0.8	69 ± 3
	5:1	14 ± 0.8	68 ± 3

**Table 3:** Storage (G′) and loss moduli(G″), and temperature of sol-gel transition (T_sol-gel_) data for different HPC-based in situ gel formulations.

Sample	G′_sol_^1^ (Pa)	G″_sol_ (Pa)	G′_gel_ (Pa)	G″_gel_ (Pa)	T_sol-gel_ (°C)
HPC 10%	5 ± 1	26 ± 3	94 ± 4	29 ± 3	45.8 ± 0.3
HPC 12%	26 ± 2	76 ± 4	211 ± 14	88 ± 4	46.1 ± 0.4
HPC 15%	160 ± 14	327 ± 22	747 ± 32	270 ± 10	46.2 ± 0.4
HPC 10% + K_2_SO_4_	12 ± 2	57 ± 3	180 ± 12	89 ± 8	34.2 ± 0.3
HPC 12% + K_2_SO_4_	32 ± 2	81 ± 4	246 ± 13	117 ± 12	34.6 ± 0.3
HPC 15% + K_2_SO_4_	167 ± 11	343 ± 22	940 ± 35	346 ± 15	33.4 ± 0.2

^1^ For pure HPC samples, G′ and G″ values were determined at 35 and 50 °C; for HPC + K_2_SO_4_ samples, G′ and G″ values were determined at 25 and 37 °C, respectively.

**Table 4:** Equieffective concentrations (IC_50_) values of free and formulated CBD against MCF-7 cell lines.

Sample	IC_50_ (mg/L)	IC_50_ (µM)
CBD (as solution in ethanol)	3.36	10.7
PEO_113_-*b*-P(CyCL_1_-*co*-CL_27_)_28_/CBD-loaded micelles	3.98	12.7
HPC gels + 1 g/L PEO_113_-*b*-P(CyCL_1_-*co*-CL_27_)_28_/CBD-loaded micelles	5.19	16.5
HPC gels + 5 g/L PEO_113_-*b*-P(CyCL_1_-*co*-CL_27_)_28_/CBD-loaded micelles	5.44	17.3

## Data Availability

The data presented in this study are available in article and Supplementary Materials.

## Supplementary Materials

The following supporting information can be downloaded at: https://www.mdpi.com/article/10.3390/ijms242216534/s1.

## References

[1] Wichterle O., Lím D. (1960). Hydrophilic Gels for Biological Use. Nature.

[2] Jeong B., Kim S.W., Bae Y.H. (2002). Thermosensitive sol-gel reversible hydrogels. Adv. Drug. Deliv. Rev..

[3] Van Tomme S.R., Storm G., Hennink W.E. (2008). In situ gelling hydrogels for pharmaceutical and biomedical applications. Int. J. Pharm..

[4] Liow S.S., Dou Q., Kai D., Karim A.A., Zhang K., Xu F., Loh X.J. (2016). Thermogels: In Situ Gelling Biomaterial. ACS Biomater. Sci. Eng..

[5] Alonso J.M., Andrade del Olmo J., Perez Gonzalez R., Saez-Martinez V. (2021). Injectable Hydrogels: From Laboratory to Industrialization. Polymers.

[6] Mathew A.P., Uthaman S., Cho K.H., Cho C.S., Park I.K. (2018). Injectable hydrogels for delivering biotherapeutic molecules. Int. J. Biol. Macromol..

[7] Thambi T., Li Y., Lee D.S. (2017). Injectable hydrogels for sustained release of therapeutic agents. J. Control. Release.

[8] Xu Y., Yang M., Ma Q., Dia X., Wu G. (2021). A bio-inspired fluorescent nano-injectable hydrogel as a synergistic drug delivery system. New J. Chem..

[9] Patel M., Moon H.J., Jung B.K., Jeong B. (2015). Microsphere-Incorporated Hybrid Thermogel for Neuronal Differentiation of Tonsil Derived Mesenchymal Stem Cells. Adv. Healthc. Mater..

[10] Payyappilly S., Dhara S., Chattopadhyay S. (2014). Thermoresponsive biodegradable PEG-PCL-PEG based injectable hydrogel for pulsatile insulin delivery. J. Biomed. Mater. Res. A.

[11] Zubik K., Singhsa P., Wang Y., Manuspiya H., Narain R. (2017). Thermo-responsive poly(N-isopropylacrylamide)-cellulose nanocrystals hybrid hydrogels for wound dressing. Polymers.

[12] Zheng B.D., Ye J., Yang Y.C., Huang Y.Y., Xiao M.T. (2022). Self-healing polysaccharide-based injectable hydrogels with antibacterial activity for wound healing. Carbohydr. Polym..

[13] Klouda L. (2015). Thermoresponsive hydrogels in biomedical applications: A seven-year update. Eur. J. Pharm. Biopharm..

[14] Csóka G., Gelencsér A., Makó A., Marton S., Zelkó R., Klebovich I., Antal I. (2007). Potential application of Metolose in a thermoresponsive transdermal therapeutic system. Int. J. Pharm..

[15] Miao L., Hu J., Lu M., Tu Y., Chen X., Li Y., Lin S., Li F., Hu S. (2016). Alkynyl-functionalization of hydroxypropyl cellulose and thermoresponsive hydrogel thereof prepared with P(NIPAAm-co-HEMAPCL). Carbohydr. Polym..

[16] Gosecki M., Setälä H., Virtanen T., Ryan A.J. (2021). A facile method to control the phase behavior of hydroxypropyl cellulose. Carbohydr. Polym..

[17] Joshi S.C. (2011). Sol-Gel Behavior of Hydroxypropyl Methylcellulose (HPMC) in Ionic Media Including Drug Release. Materials.

[18] Scuderi C., Filippis D.D., Iuvone T., Blasio A., Steardo A., Esposito G. (2009). Cannabidiol in medicine: A review of its therapeutic potential in CNS disorders. Phytother. Res..

[19] Seltzer E.S., Watters A.K., MacKenzie D., Granat L.M., Zhang D. (2020). Cannabidiol (CBD) as a promising anti-cancer drug. Cancers.

[20] Millar S.A., Maguire R.F., Yates A.S., O’Sullivan S.E. (2020). Towards Better Delivery of Cannabidiol (CBD). Pharmaceuticals.

[21] Momekova D., Ivanov E., Konstantinov S., Ublekov F., Petrov P.D. (2020). Nanocomposite cryogel carriers from 2-hydroxyethyl cellulose network and cannabidiol-loaded polymeric micelles for sustained topical delivery. Polymers.

[22] Chountoulesi M., Selianitis D., Pispas S., Pippa N. (2023). Recent Advances on PEO-PCL Block and Graft Copolymers as Nanocarriers for Drug Delivery Applications. Materials.

[23] Grancharov G., Atanasova M.D., Aluani D., Yoncheva K., Tzankova V., Trusheva B., Forys A., Trzebicka B., Petrov P.D. (2020). Functional block copolymers bearing pendant cinnamyl groups for enhanced solubilization of caffeic acid phenethyl ester. Polym. J..

[24] Atanasova M.-D., Grancharov G., Petrov P.D. (2021). Poly(ethylene oxide)-*block*-poly(α-cinnamyl-ε-caprolactone-*co*-ε-caprolactone) diblock copolymer nanocarriers for enhanced solubilization of caffeic acid phenethyl ester. J. Polym. Sci..

[25] Gil E.S., Hudson S.M. (2004). Stimuli-reponsive polymers and their bioconjugates. Prog. Polym. Sci..

[26] Pencheva V., Margaritova E., Borinarova M., Slavkova M., Momekova D., Petrov P.D. (2018). A novel approach for fabricating nanocomposite materials by embedding stabilized core-shell micelles into polysaccharide cryogel matrix. Carbohydr. Polym..

[27] Fraguas-Sánchez A.I., Fernández-Carballido A., Simancas-Herbada R., Martin-Sabroso C., Torres-Suárez A.I. (2020). CBD loaded microparticles as a potential formulation to improve paclitaxel and doxorubicin-based chemotherapy in breast cancer. Int. J. Pharm..

[28] D’Aloia A., Ceriani M., Tisi R., Stucchi S., Sacco E., Costa B. (2022). Cannabidiol Antiproliferative Effect in Triple-Negative Breast Cancer MDA-MB-231 Cells Is Modulated by its Physical State and by IGF-1. Int. J. Mol. Sci..

[29] Nie S., Hsiao W.L., Pan W., Yang Z. (2011). Thermoreversible Pluronic F127-based hydrogel containing liposomes for the controlled delivery of paclitaxel: In vitro drug release, cell cytotoxicity, and uptake studies. Int. J. Nanomed..

[30] (2017). Biological Evaluation of Medical Devices—Part 5: Tests for In Vitro Cytotoxicity.

[31] Mosmann T. (1983). Rapid colorimetric assay for cellular growth and survival: Application to proliferation and cytotoxicity assays. J. Immunol. Methods.

