# Supplementary material for: In situ Gelling Hydroxypropyl Cellulose Formulation Comprising Cannabidiol-Loaded Block Copolymer Micelles for Sustained Drug Delivery
Source: Int J Mol Sci. 2023 Nov 20;24(22):16534. doi: 10.3390/ijms242216534 (PMC10671718; doi:10.3390/ijms242216534)
Supplement: Supplementary file 1 [file ijms-24-16534-s001.zip › ijms-2696850-supplementary.pdf]

Electronic Supplementary Information

In situ Gelling Hydroxypropyl Cellulose Formulation Comprising Cannabidiol-Loaded Block Copolymer Micelles for Sustained Drug Delivery

Katya Kamenova[1], Denitsa Momekova[2], Georgy Grancharov[1], Anna Prancheva[1], Natalia Toncheva-Moncheva[1], Ervin Ivanov [2,3], Spiro Konstantinov [2], and Petar D. Petrov [1,*]

[1] Institute of Polymers, Bulgarian Academy of Sciences, 1113 Sofia, Bulgaria; kkamenova@polymer.bas.bg (K.K.); granchar@polymer.bas.bg (G.G.); a_prancheva@polymer.bas.bg (A.P.); ntoncheva@polymer.bas.bg (N.T.)

[2] Faculty of Pharmacy, Medical University of Sofia, 1000 Sofia, Bulgaria; dmomekova@pharmfac.mu-sofia.bg (D.M.); ervin.ivanov@gmail.com (E.I.); skonstantinov@pharmfac.mu-sofia.bg (S.K.)

[3] Pobelch Gle Ltd., Byalo Pole St. 3, 1618 Sofia, Bulgaria

* Correspondence: ppetrov@polymer.bas.bg; Tel.: +359-2-9796335

EXPERIMENTAL

Synthesis of diblock copolymers

Poly(ethylene oxide)-*block*-poly(*α*-cinnamyl-ε-caprolactone-*co*-ε-caprolactone) diblock copolymers was synthesized via two-step procedure involving ring-opening polymerization of ε-CL and *α*-propargyl-ε-caprolactone from methoxy poly(ethylene oxide) macroinitiator and subsequent grafting of cinnamyl groups via "click" reaction **[1]**.

Synthesis of poly(ethylene oxide)-block-poly(α-propargyl-ε-caprolactone-co-ε-caprolactone)

Polymerization was carried out in an oven dried 50 ml two-neck round bottom flask under an inert atmosphere. In a typical run, methoxy PEO_{113}-OH (1.5 g, 0.3 mmol, 1 eq.) dried by azeotropic distillation from anhydrous toluene, ε-CL (1.08 g, 9.45 mmol, 90 eq.), α-propargyl-ε-caprolactone (0.16 g, 1.05 mmol, 10 eq.), and toluene (15 mL) were placed in the flask. The solution was degassed by argon flow for 20 min and the catalyst $Sn(Oct)_2$ (21.5 mg, 0.053 mmol, 0.5 mol % of both monomers) was added, followed by further degassing for 20 min. The reaction mixture was stirred at 115 °C for 24 hours and poured into cold isopropanol. The precipitated copolymer was collected by filtration and dried thoroughly in vacuum. Yield 2.5 g. ^1H NMR(600 MHz, $CDCl_3$) δ (ppm)= 4.21 (t, 2H, -CH_2O-), 4.05 (t, 54H, -CH_2O-), 3.50-3.82 (m, 450H, -CH_2O-), 3.37 (s, 3H, CH_3-O-), 2.53-2.58 (m, 1H, COC*H*CH_2), 2.43-2.49 (m, 1H, COCHCH_2), 2.35-2.42 (m, 1H, COCHCH_2), 2.30 (t, 54H, -COCH_2O-), 2.00 (2, 1H, -C≡CH-), 1.50-1.79 (m, 108H, CH_2-CH_2-CH_2-CH_2-O-), 1.30-1.48 (m, 54H, COCH_2CH_2).

Synthesis of poly(ethylene oxide)-block-poly(α-cinnamyl-ε-caprolactone-co-ε-caprolactone)

In an oven dried 50 ml two-neck round bottom flask the copolymer (4.3 % propargyl units; 1g, 0.124 mmol, 1 eq.), CuI (109.7 mg, 0.576 mmol, 4 eq.), and cinnamyl azide (45.8 mg, 0.288 mmol, 2 eq.) were added under an inert atmosphere, and degassed 3 times. After that, 5 ml of THF was added and the system was purged for approximately 20 min with inert gas, parallel to a vial of N,N-diisopropylethylamine. Finally, degassed DIPEA (74.4 mg, 0.576 mmol, 4 eq.) was added, the whole system was purged additionally with an inert gas for 20 min and the mixture was stirred at temperature 40°C for 24 h. Then, 50 ml of THF was added and the reaction mixture was passed through a neutral aluminium oxide (Al_2O_3) plug to remove the copper salts. The mixture was then dissolved in a minimal amount of THF and precipitated into cold isopropanol. Yield 0.98 g. ^1H NMR(600MHz, CD_2Cl_2)) δ (ppm)= 7.18-7.60 (m, 6H, Ar*H*), 4.21 (t, 2H, -CH_2O-), 4.05 (t, 54H, -CH_2O-), 3.45-3.95

(m, 450H, -CH_2O-), 3.40 (s, 3H, CH_3-O-), 2.26-2.38 (m, 54H, -COCH_2O-), 1.55-1.80 (m, 108H, CH_2-CH_2-CH_2-CH_2-O-), 1.33-1.46 (m, 54H, $COCH_2$CH_2).

Synthesis of poly(ethylene oxide)-block-poly(ε-caprolactone)

Polymerization was carried out in an oven dried 50 ml two-neck round bottom flask under an inert atmosphere. In a typical run, PEG_{113}-OH (1.5 g, 0.3 mmol,1 eq.) dried by azeotropic distillation from anhydrous toluene, ε-CL (1.2 g, 10.5 mmol, 35 eq.) and toluene (15 mL) were placed in the flask. The solution was degassed by argon flow for 20 min and catalyst $Sn(Oct)_2$ (21.5 mg, 0.053 mmol, 0.5 mol % of the monomer) was added, followed by further degassing for 20 min. The reaction mixture was stirred at 115 °C for 24 hours and poured into cold isopropanol. The precipitated copolymer was collected by filtration and dried thoroughly in vacuum. Yield 2.6 g. ^{1}H NMR(600 MHz, $CDCl_3$) δ (ppm)= 4.22 (t, 2H, -CH_2O-), 4.05 (t, 57H, -CH_2O-), 3.50-3.85 (m, 450H, -CH_2O-), 3.37 (s, 3H, CH_3-O-), 2.31 (t, 57H, -COCH_2O-), 1.55-1.80 (m, 114H, CH_2-CH_2-CH_2-CH_2-O-), 1.33-1.43 (m, 57H, $COCH_2$CH_2).

Table S1. *Composition and molecular characteristics of diblock copolymers.*

Copolymer	$M_n^{(NMR)}$ (g/mol)	$M_n^{(SEC)}$ (g/mol)	$DI^{(SEC)}$
PEO_{113}-b-P($CyCL_1$-co-CL_{27})$_{28}$	8400	6200	1.04
PEO_{113}-*b*-P($CyCL_1$-*co*-CL_{12})$_{13}$	6680	5100	1.07
PEO_{113}-*b*-PCL_{29}	8310	7950	1.12

[1] Atanasova, M-D, Grancharov, G, Petrov, PD. Poly(ethylene oxide)-*block*-poly(α-cinnamyl-ε-caprolactone-*co*-ε-caprolactone) diblock copolymer nanocarriers for enhanced solubilization of caffeic acid phenethyl ester. *J Polym Sci*. 2021; 59: 251–260.

FIGURES

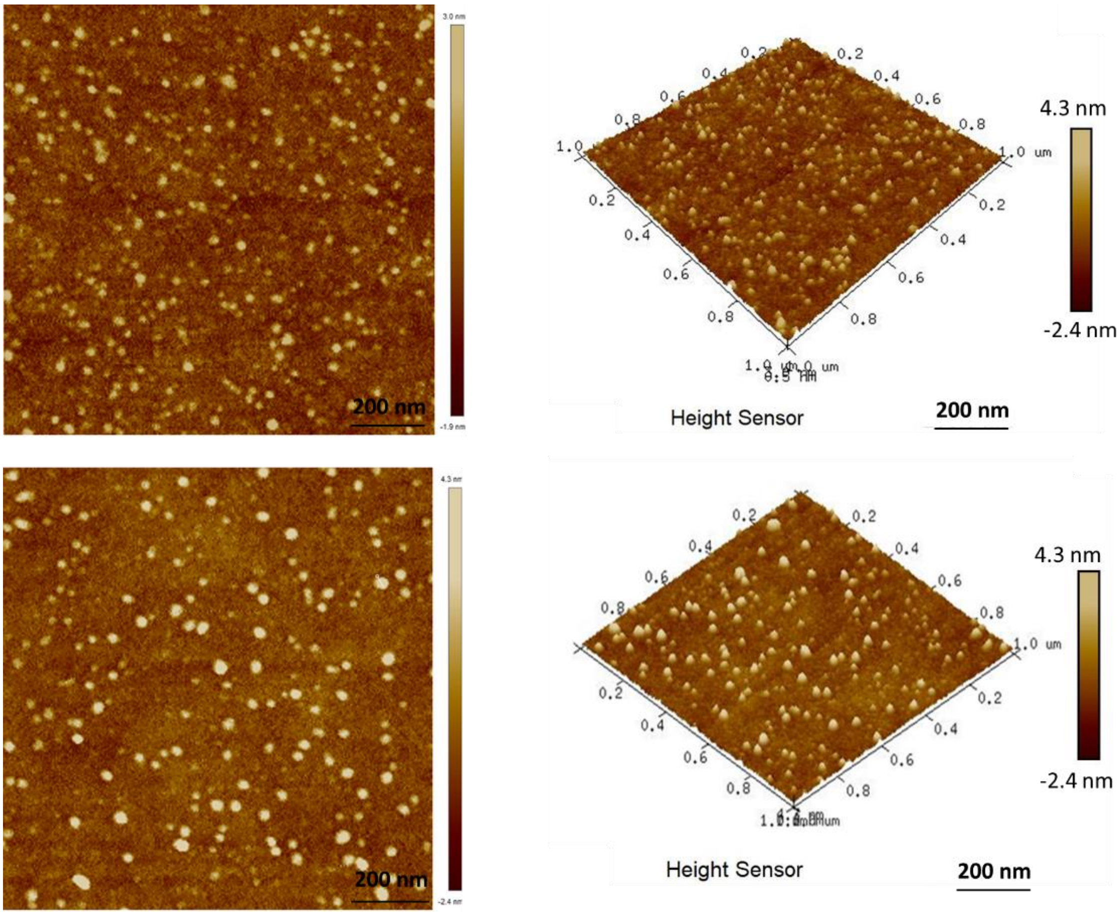

Figure S1. Representative 2D and 3D AFM images of blank (top) and CBD-loaded PEO$_{113}$-*b*-P(CyCL$_1$-*co*-CL$_{27}$)$_{28}$ micelles (bottom).

Pure HPC

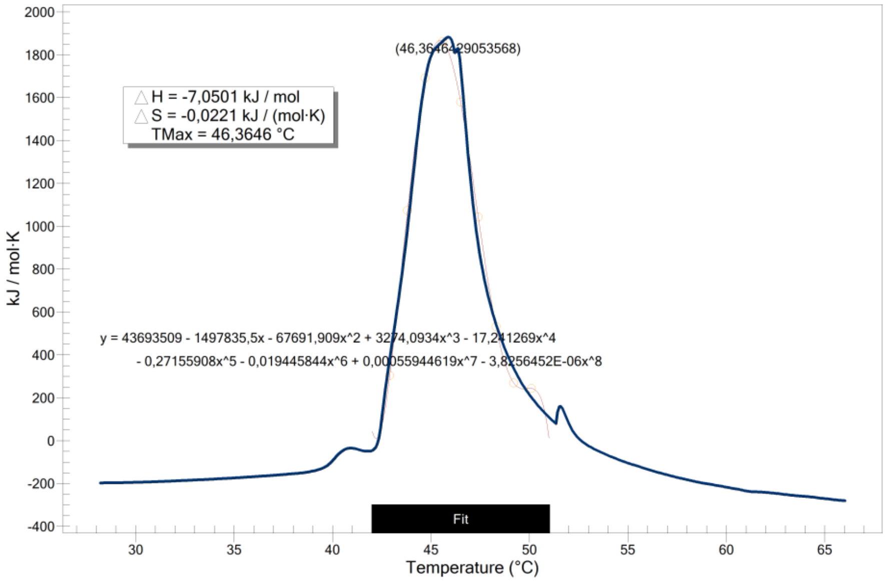

HPC + K_2SO_4

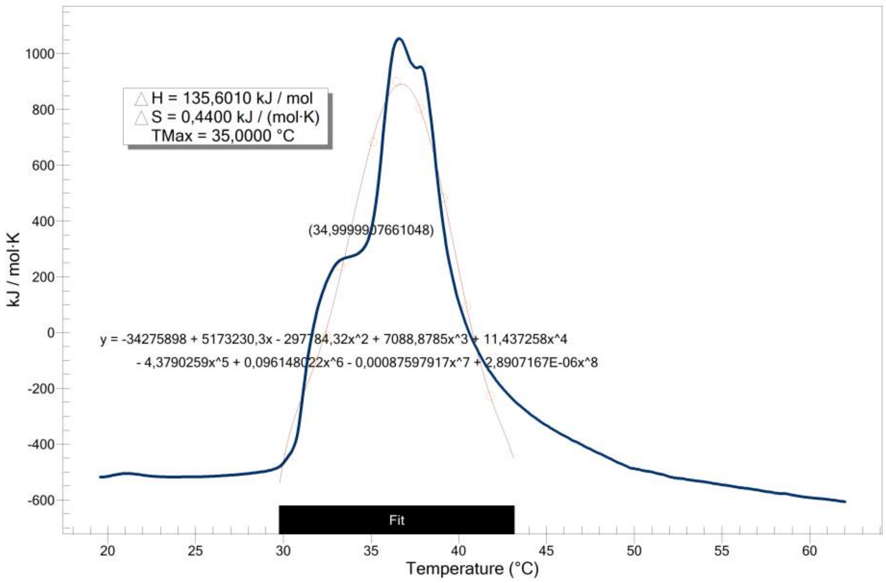

Figure S2. NanoDSC plots of aqueous HPC solutions (5 mass %), without (top) and with 0.15 molL^{-1} K_2SO_4 (bottom).

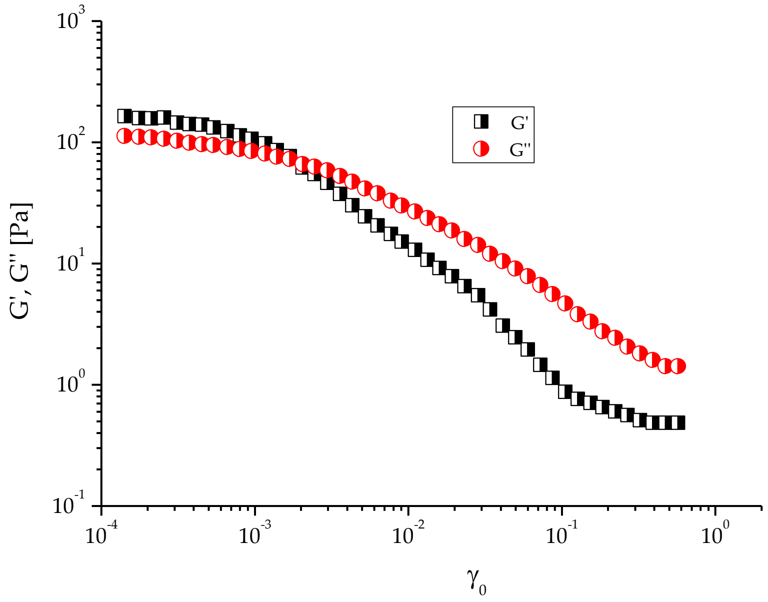

Figure S3. Variation in storage (G′) and loss (G′′) moduli as a function of the strain amplitude for HPC (10 mass %) hydrogel containing K_2SO_4 (0.15 molL^{-1}). The oscillation amplitude sweep tests were carried out at 37 °C at a frequency of 1 Hz in the γ_0 range from 0.0001 to 1.

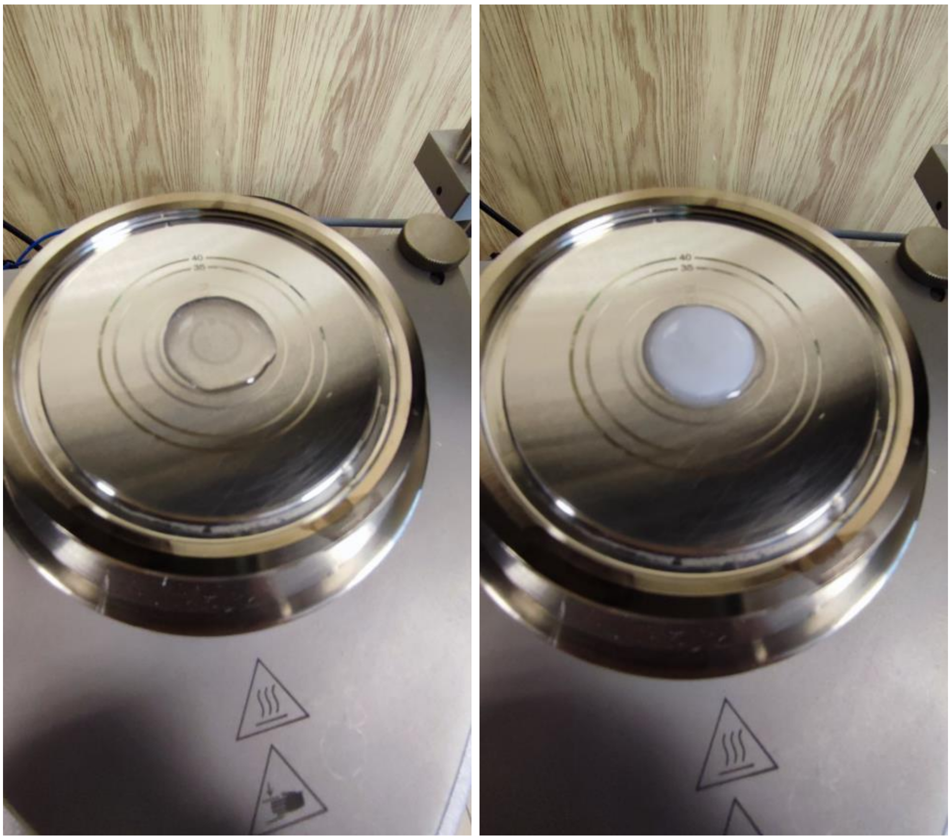

Figure S4. Digital images of an aqueous HPC (10 mass %) solution, containing K_2SO_4 (0.15 $molL^{-1}$) and CBD-loaded micelles (1 gL^{-1}) at 25 ^oC (left), and the corresponding hydrogel formed at 37 ^oC (right).

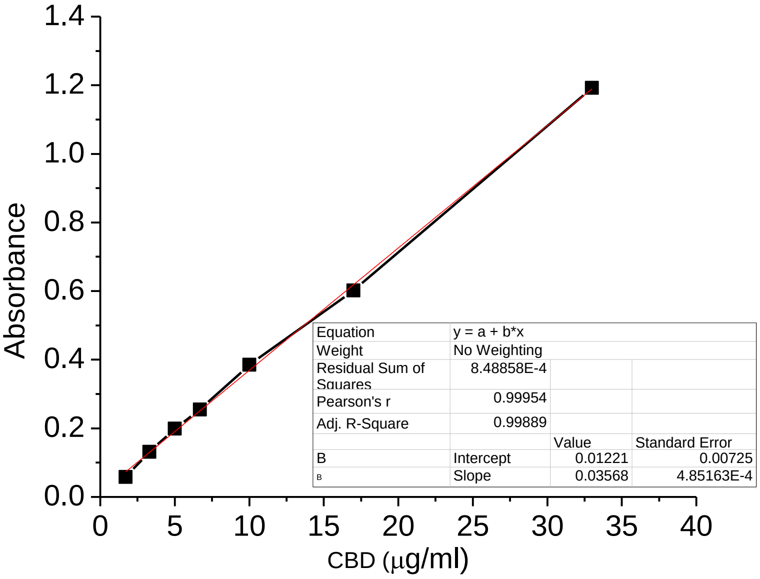

Figure S5. Standard curve of CBD in methanol at λ= 274 (R^2 = 0.99954).

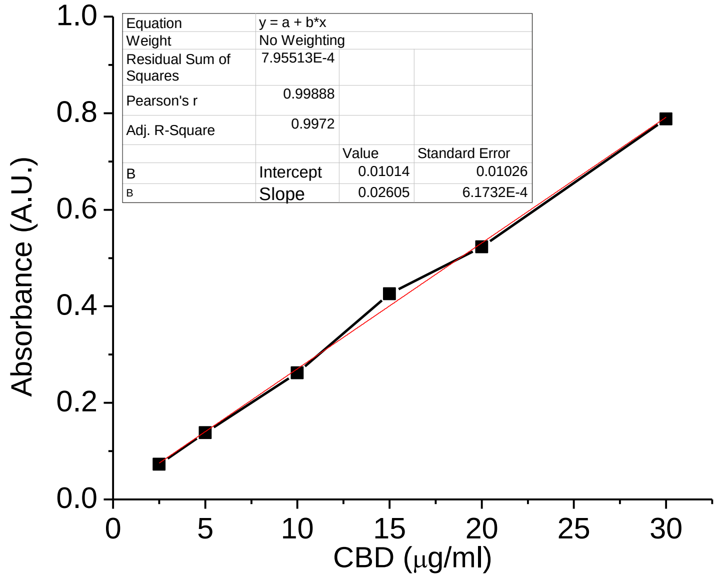

Figure S6. Standard curve of CBD in PBS + 5% ethanol at λ= 274 (R^2=0.9988).
